# Knowledge, Attitudes and Practice of Iranian Medical Specialists regarding Hepatitis B and C

**Published:** 2010-09-01

**Authors:** Ali Kabir, Seyed Vahid Tabatabaei, Siamak Khaleghi, Shahram Agah, Amir Hossein Faghihi Kashani, Mehrdad Moghimi, Fahimeh Habibi Kerahroodi, Seyed-e-Hoda Alavian, Seyed Moayed Alavian

**Affiliations:** 1 Nikan Health Researchers Institute, Gastrointestinal and Liver Diseases Research Center, Iran University of Medical Sciences, Tehran, Iran; 2 Baqiyatallah Research Center for Gastroenterology and Liver Disease, Baqiyatallah University of Medical Sciences, Tehran, Iran; 3 Gastrointestinal and Liver Diseases Research Center, Iran University of Medical Sciences, Tehran, Iran; 4 Department of Surgery, Shahid Beheshti University of Medical Sciences, Tehran, Iran

**Keywords:** Hepatitis B, Hepatitis C, Health Knowledge, Attitudes, Practices, Iran

## Abstract

**Background and Aims:**

Health care workers (HCWs) are at risk of contracting and spreading hepatitis B virus (HBV) and  hepatitis C virus (HCV) to others. The aim of this study was to evaluate knowledge, attitudes and behavior of physicians concerning HBV and HCV.

**Methods:**

A 29-item questionnaire (reliability coefficient = 0.7) was distributed at two national/regional congresses and  two university hospitals in Iran. Five medical groups (dentists, general practitioners, paraclinicians, surgeons and internists) received 450 questionnaires in 2009, of which 369 questionnaires (82%) were filled out.

**Results:**

Knowledge about routes of transmission of HBV and HCV, prevalence rate and seroconversion rates secondary  to a needlestick injury was moderate to low. Concern about being infected with HBV and HCV was 69.4±2.1 and 76.3±2 (out of 100), respectively. Complete HBV vaccination was done on 88.1% of the participants. Sixty percent had checked their hepatitis B surface antibody (anti-HBs), and 83.8% were positive. Only 24% of the surgeons often used double gloves and 28% had reported a needlestick. There was no significant correlation between the different specialties and: concern about HBV and HCV; the underreporting of needlestick injuries; and correct knowledge of post-needlestick HBV infection.

**Conclusions:**

Although our participants were afraid of acquiring HBV and HCV, knowledge about routes of transmission,  prevalence, protection and post-exposure seroconversion rates was unsatisfactory. By making physicians aware of possible post-exposure prophylaxis, the underreporting of needlestick injuries could be eliminated. Continuous training about HBV and HCV transmission routes, seroconversion rates, protection, as well as hepatitis B vaccination and checking the anti-HBs level, is a matter of necessity.

## Introduction

Infection with hepatitis B virus (HBV) and  hepatitis C virus (HCV) is a problem of worldwide significance [1]. While hepatitis B is vaccine-preventable [2], HCV infection has no effective vaccine [3]. According to a World Health Organization (WHO) estimate, two billion people in the world have serological evidence of prior HBV infection [4], and up to 3% (170 million) are infected with HCV [5]. HBV and HCV can lead to cirrhosis and hepatocellular carcinoma [6]. HCV is a leading cause of end-stage liver disease and the most common indication for liver transplantation [2]. Although HCV has been less prevalent since the 1990s in the western world, it is still endemic in some African and Asian countries [6].

Of the world’s carriers of HBV, 75% are from  Asia [2], and Iran represents a low-to- moderate category [7]. Our recent systematic reviews in Iran between 2001 and 2007 found a prevalence of HBV and HCV of 2.14% and 0.16%, respectively [7][8]. Iranian studies show that 1.3 to 8.69% of the population are chronic HBV carriers [9]. The epidemiology of infection is also changing from a vertical to a horizontal route [10]. HBV prevalence has decreased dramatically in Iran, and it is classified as of low endemicity now. This may be due to an improvement of people’s knowledge of HBV risk factors, the national vaccination program, since 1993, for all neonates, and the vaccination of high-risk groups [10]. Coverage reached 94% by 2005 [10]. Although there is no mandatory HBV vaccination for  health care workers (HCWs) in Iran, it is mandatory for neonates and adolescents.

HBV and HCV have common routes of  transmission, such as occupational exposure among HCWs, unprotected sexual contact, vertical transmission, intravenous drug use [11][12] or through blood products and contamination during medical procedures [2].

The prevalence of hepatitis C is remarkable in  high-risk groups such as thalassemics [13], intravenous drug users [2], and chronic hemodialysis patients [11].

For the most part, surgeons in the United States  (1998) seldom reported needlestick injuries and rarely used double gloves [14]. Another study indicated that 4% and 61% of HCWs, respectively, were unaware that HBV and HCV can be transmitted by needlestick injuries [15].

Iranian midwifery students and graduates [16] and surgeons [17] had an undesirable level of knowledge of HBV [16]. However, dentists were found to have a good knowledge of HBV [18].

It is notable that some other studies in Iran had evaluated knowledge or behavior of groups other than HCWs, or physicians, in dealing with HBV [19][20]. Few studies have investigated hepatitis C specifically in Iran. Despite the decrease in HBV prevalence in our country, there has been no decrease in hepatitis C [2].

It has been demonstrated that patient medical histories are unreliable in identifying exposure to HBV infection [17]. The needlestick injuries are a personal, legal and professional hazard to HCWs, physicians and their patients [21]. As hepatitis B and C can lead to much morbidity and mortality, and physicians and other medical specialists are a high-risk group for acquiring HBV and HCV infection and for transmission to their patients and close contacts, it makes sense to evaluate physicians’ knowledge, attitudes and practice not only towards HBV transmission and protection, but also towards HCV, to which little attention has yet been paid.

## Materials and Methods

### Questionnaire

We designed a 29-item self-administered  questionnaire assessing risk of transmission; seroconversion rates; the actual prevalence of HBV and HCV in Iran; vaccination against HBV; use of double gloves, and protective eyewear; rate of needlestick injury and its report; checking the status of viral hepatitis; using disposable syringes and discarding them; and post-exposure prophylaxis. It consisted of use of the likert scale; yes/no and a few open-ended questions, in addition to some demographics (age, gender, specialty, work time per week). For validating the questionnaire, some physicians, expert in standardizing questionnaires, confirmed the face and content validity of the primary questionnaire, and some questions were omitted according to their comments. According to our pilot study on 60 randomly selected physicians, the reliability coefficient of the questionnaire was 0.7, using Cronbach’s alpha. The reliability coefficients of each of the three parts of the questionnaire for knowledge, attitude and practice issues were 0.65, 0.75, and 0.67. The Ethical Committee of Baqyitallah Research Center for Gastroenterology and Liver Disease, Tehran, Iran approved the proposal.

### Samples

We distributed questionnaires among participants in two viral hepatitis congresses in advance, in Tehran (capital of Iran, a national congress) and in Zanjan (in western Iran, a regional congress) and also among physicians of two university hospitals in Ahvaz (in southern Iran). Participants retuned 370 (82%) of the 450 distributed questionnaires. We excluded one questionnaire due to its missing more than 50% of the data. Considering our previous paper about a Knowledge, attitudes and practice (KAP) survey concerning bloodborne diseases in surgeons [17], we estimated that the average percentage of physicians with a sufficient score for all knowledge, attitudes and practices was about 19%. We used a rate-estimation formula for calculating sample size, while we assumed the precision of this ratio to be 0.04% and type-one error equal to 0.05. Considering a response rate of almost 80%, the total sample size was calculated as 450.

In this cross sectional study, we considered dentists, general practitioners (GPs), paraclinicians (laboratory specialists, pathologists, anesthesiologists, radiologists, and parasitologists), surgeons  (gynecologists, general surgeons, and orthopedic surgeons) and internists (internal medicine specialists, pediatricians, cardiologists, infectious disease specialists, dermatologists, and emergency medicine specialists) as different groups.

### Data Analysis

We used mean±SE (standard error), t-test, one  way ANOVA with post hoc, Kruskal-Wallis, and chi-square in analysis by SPSS 13 software (SPSS Inc. Chicago, Illinois, USA). The authors considered differences and correlations, with P < 0.05 as statistically significant.

## Results

Of 369 participants who returned completed  forms, 13 cases were not active in medical practice. Therefore, analysis was done on the 356 remaining cases, with 166 (46.6%) women, 157 (44.1%) men and 33 (9.3%) who did not state their gender. They had a mean age of 41.1±.5 (range: 25-79) years, a median duration in medical practice of 12 (range: 0.3-50) years, and a median weekly medical practice of 35 (range: 2-120) hours. Of participants, 36.3%  were employees of university hospitals and clinics of the Ministry of Health, 39.8% worked in private practice, and 23.9% worked in both private practice and as employees of clinics and university hospitals.

Only 44.9% were aware that viral hepatitis B and C can be transmitted from patients to patients, dentists to patients and vice versa. Others believed that at least one of these routes could not be a possible risk of transmission.

### Knowledge

Participants estimated that 11.3±1.1 and 8.9±1.4  of their patients were positive for HBV and HCV, respectively.

Knowledge about HBV and HCV routes of transmission, prevalence rate and seroconversion rates secondary to a needlestick injury was unsatisfactory (Table 1). Most participants were not aware and most of them underestimated the seroconversion rates, post-exposure to HBV or HCV.

**Table 1:** Correct knowledge about routes of transmission, prevalence rate and seroconversion rates secondary to an HBV and HCV needlestick injury.^a^

	**Yes**	**No**	**No idea**	**Correct answer**	**Reference**
Transmission is possible via saliva, %	53.3	28.7	18	Yes	[22]
Transmission is possible via dentistry, %	86	1.1	12.9	Yes	[23]
Transmission after needlestick is higher for HBV in comparison with HIV , %	71.1	8.1	20.8	Yes	[24]
Prevalence of HBV is lower than 1% in Iranian population, %	14.9	66.9	18.2	2.14	[7]
Prevalence of HCV is lower than 1% in Iranian population, %	47.2	32.6	20.2	0.16	[7]
Using glove is useful in preventing infection with HBV , %	83.7	3.1	13.2	Yes	[17]
Correct knowledge about seroconversion rate secondary to an HBV needlestick injury, %	11.8	33.7	54.5	25-30	[24]
Correct knowledge about seroconversion rate secondary to an HCV a needlestick injury, %	14.3	28.7	57	3-10	[24]

^a^ HBV: hepatitis B virus; HCV: hepatitis C virus; HIV: human immunodeficiency virus

According to table 1, 47.2% versus 66.9% had a correct estimation of HCV (<1%) and HBV (>1%) prevalence, respectively. However, 2.8% of our participants estimated HCV prevalence in their patients correctly (between 0.1-0.3%), 1.1% underestimated it (<0.1%), 47.2% overestimated it (>0.3%), and 48.9% had no idea. These percentages were 14.3% (correct answer; between 2-3%), 13.8% (underestimated; <2%), 36.5% (overestimated; > 3%), and 35.4% (no idea) for HBV.

### Attitudes

Participants requested HBV and HCV screening  tests for 30.1±2.5 and 20.9±2.6 percent of their patients, respectively.

Concern about being infected with HBV and HCV was 69.4±2.1 and 76.3±2 (of 100 total score), respectively.

### Practice

Among 61.3% who did surgical procedures, only  24% often, and none always, used double gloves; while 43.8% of them always used glasses and/or masks.

Complete vaccination against hepatitis B was done on 88.1% of the participants. In addition, 8.1% had insufficient vaccination (less than 3 times), and 3.9% of them were in process. Merely 60% of the participants (210 cases) had checked their hepatitis B surface antibody (anti-HBs) level, of whom 83.8% were positive.

Of the participants, 3.6% did not use discarded syringes. Rate of covering (always or often) the needle before discard was 73.4%.

While 52.1% had scratches, ulcers or open wounds on their hands most of the time (sometimes, often and always) and 47.1% had at least one needlestick per year, just 28% had always/often reported their actual needlestick injury and 40% did not.

They had experienced 1.9±.4 times needlestick injuries over the last 3 years and were stuck by a needle while treating a patient positive for HBV and HCV in 5.1% and 1.2% of cases, respectively.

Contamination of their eyes and mucosa by the secretions of a patient positive for HBV and HCV was reported by 3.7% and 0.8% of them, respectively.

Nearly all cases with the reported needlestick injury had been treated with hepatitis B vaccine and/ or immunoglubolin.

Completion of vaccination was strictly related to participants’ knowledge about viral hepatitis prevalence. Those who were not vaccinated against HBV had a significantly falsely higher estimation of HBV (55.9±7.4 vs. 39.6±2.5, P = .043) and HCV (72.6±6.9 vs. 49.6±2.7, P = .003) infection in their patients.

### Specialty-related analysis

Dentists and general practitioners were younger and less experienced in comparison with paraclinicians and internists, who were older and more experienced (P < 0.001 and P = 0.017, respectively).

While general practitioners were more active, dentists worked less (P = 0.029). Surgeons requested more while dentists requested fewer laboratory tests for HBV (P < 0.001) and HCV (P = 0.017) infection in their patients (Table 2).

**Table 2:** Specialty characteristics and subgroup analysis^a^

	**Dentist**	**General practitioner**	**Para-clinician**	**Surgeon**	**Internist**	**P-value**
Percent of participants	11.8	34.8	15.2	19.6	18.6	<.001 b
Mean age± SE, years	37.3±1.4	38.6±.9	45.1±1.7	41.6±1.3	45.1±1.6	<.001^c^
Mean work experience ±SE, year	11.4±1.3	11.4±.94	15.7±1.9	12.5±1.3	16.4±1.7	.017 c
Mean work per week ±SE, hour	27.8±3	43.2±3.1	34.8±3.2	32.9±3.4	37.4±2.6	.016 c
Coverage of HB vaccination, %	100	84.1	77.4	94.7	94.6	.025 b
Lab request for HBV infection in their patients, %±SE	3.8±2	13.7±3.1	34.9±13.1	63.4±7.7	26.7±6.7	<.001 b
Lab request for HCV infection in their patients, %±SE	4.2±2.4	9.1±2.7	26.2±14.3	43.8±10.3	24.8±7.2	.017 b
Correct knowledge about transmission of HBV and HCV via saliva, %	75	59.2	32.3	57.5	65.8	.001 b
Check titer of anti-HBs Ab, %	75	49.3	64.5	60	78.4	.046 b
Positive titer of anti-HBs Ab, %	66.7	32.4	61.3	55	73	.037 b
Often usage of mask during work, %	100	29.4	50	47.2	27.8	<.001 b
Wear two gloves during work, %	33.3	27.5	10	32.5	7.9	<.001 b

^a^ HBV: hepatitis B virus; HCV: hepatitis C virus; anti-HBs Ab: hepatitis B surface antibody

^b^ Chi-square test

^c^ One way ANOVA

Dentists, paraclinicians and surgeons estimated about 60% of their patients being infected with HCV, while it was about 39% and 37% for internists and general practitioners, respectively (P = 0.024).

In descending rank, the percentage of needlestick injuries was 6.8±3 in surgeons, 2.5±1.5 in paraclinicians, 1.7±0.3 in dentists, 0.9±0.2 in general practitioners and 0.45+0.1 in internists.(P < 0.001) The mean percentage of needlestick injuries was 60.6%, 48.6%, 48.4%, 45.8% and 27.5% among general practitioners, internists, paraclinicians, dentists, and surgeons, respectively.(P = 0.024)

Coverage of HB vaccination had a statistically significant difference among different specialties (P = 0.025); it was highest among dentists and lowest in paraclinicians (Table 2).

Dentists were more alert to possible HBV and HCV transmission via saliva (P = 0.001). Not only had internists checked titer of anti-HBs Ab more than others (P = 0.046), but also had higher positive titer themselves as well (P = 0.037). Usage of mask and two gloves were both more common among dentists (P < 0.001 in both) (Table 2).

There was no significant correlation between different specialties and the remaining variables.

## Discussion

In the present study, different medical specialties  overestimated the prevalence of HBV- and HCVinfected patients, while most protection measures were underutilized by them. Knowledge of the correct transmission route of HBV and HCV was unsatisfactory in most of the participants. Our study indicates that dentists had a better knowledge of HBV transmission via saliva in comparison with the other specialists. Interestingly, those who were not vaccinated against HBV had significantly falsely higher estimation of HBV and HCV infection in their patients, which demonstrate that fear is not enough to ensure the taking of protective measures. This finding is in concordance with a recent study among Iranian surgeons on blood-borne pathogens [17].

In the present study, a high percentage of complete vaccination against hepatitis B could explain a nonsignificant correlation between vaccination and most of the variables. The fact that general practitioners and surgeons checked their anti-HBs level the least is a matter of great concern. In our previous study, 76% of the surgeons had undergone complete HB vaccination [17], while in the present study it had increased to 94.7%, although the checking of anti- HBs level has not progressed significantly [17]. As general practitioners were the busiest of all, we may attribute this lack of concern to their workload. In an Italian survey, the majority of dentists were not immunized against HBV, because 42.8% considered it useless and 33.3% unsafe [25]. Fortunately, our study does not support this data. In another study, vaccination against HBV was done in 94.9% of dentists with complete vaccination in 74.8% of them and only 47.9% of them have been checked for HBV antibodies after vaccination [26]. These figures in our study have increased to 100% and 75%, respectively (Table 2).

It is notable that even in dentists with 100% coverage of HBV vaccination in our study, a positive titer of anti-HBs level was not satisfactory. Therefore, checking post-vaccination anti-HBs level should be mandatory and subjects with low anti-HBs levels measured at least one month after their last vaccine dose (< 10 mIU/mL), with a negative result of both hepatitis B surface antigen (HBsAg), and hepatitis B core antibody (anti-HBc) considered as nonresponders [24] and followed.

Participants requested more HBV screening tests for their patients. It is obvious that our participants as medical specialists took HCV less seriously. Notably, surgeons requested more lab screening tests for their patients.

Although the use of double gloves can help ensure better protection as a second barrier [27], in a recent study in Iran, 12.9% of surgeons always used double gloves [17] and in a study in the United States, surgeons rarely used double gloves [14].

In our study, double gloves were used by 33% of surgeons, which is a sign of positive progress, although it is certainly not enough. Reports from several countries indicate that some dentists do not engage in safe practices, especially in wearing gloves, facemasks, or protective eye glasses [26]. In the present study, dentists are more used to wearing double gloves than others (Table 2), although statistically no significant difference exists between surgeons and dentists in the use of double gloves. The probability of acquiring a blood-borne infection following occupational exposure, depending on the body fluid involved, and the infectivity of the patient, has been estimated at 0.5% for HCV, and 18%-30% for HBV [24].

In comparison with HCV, HBV prevalence was better known to our participants. (Table 1). Although disappointingly most of our participants did not know the correct percentage of seroconversion rate, secondary to a HBV and HCV needlestick injury (Table 2) even if 5.1% and 1.2%, respectively, of the participants mentioned that they had been stuck by a needle while treating a patient positive for HBV and HCV, and they also under reported these injuries. This underscores the depth and seriousness of the problem in the health system as to the high seroconversion rate of HBV and the lack of available vaccination for HCV [24].

Actually, it is almost a worldwide issue, as other studies show that nearly 80% of surgeons never, or rarely, report needlestick injuries [27][28][29][30][31] as well. Surgeons do not usually report their needlestick injuries [17]. Unfortunately, contamination of physicians’ eyes and mucosa by the secretions of a patient positive for HBV and HCV was only reported  by 3.7% and 0.8% of physicians, respectively, which may be due to lack of sufficient knowledge about possible post-exposure prophylaxis, or to their lack of interest in their own self preservation.

Complete vaccination and anti-HBs titer checking should be a must for all HCWs including medical specialists; and issuing a special card noting the number of vaccination shots and the last anti-HBs titer seems wise. As our participants’ health is not simply a matter of their own concern; but their infection with HBV and HCV can be dangerous for others, so their reeducation for both HBV and HCV prevention, seroconversion rates and the importance of reporting their needlestick injuries should be of paramount concern. Recommending usage of double gloves and eye protection is a good and easily modifiable behavior [22][23] and HCWs must be aware of first aid to meet the post-exposure needs as follows: for percutaneous exposure, encourage bleeding and wash with soap and water; for cutaneous contamination, wash with soap and  water; for mucous membrane contamination, flush with water. Eyes should be irrigated with clean water, saline, or sterile irrigants [24].

HCWs including physicians should be informed of the medicolegal and clinical relevance of reporting an exposure, and they must have rapid access to expert consultants for treatment and follow-up. The management of an occupational exposure to HBV differs according to the susceptibility of the exposed HCWs and the serostatus of the source. When indicated, post-exposure prophylaxis with HBV vaccine, hepatitis B immunoglobulin or both must be started as soon as possible (within 1-7 days). In the absence of prophylaxis against hepatitis C virus (HCV) infection, follow-up management of HCV exposure depends on which type of antiviral treatment during the acute phase is chosen [24].

We recommend observing the behavior of physicians and other HCWs toward an HBV- or HCV-infected patient in future studies as unfriendly behavior may cause patients to conceal the truth, leading to unwanted spread of disease.

We also recommend that the production of undisposable syringes be stopped in order to prevent unintentional reusage of syringes by HCWs and intentional reusage by IV drug abusers.

There were a few limitations and probably a few confounding factors in this study. For instance we cannot conclude that dentists avoid requesting HBV and HCV lab tests for their suspected patients, as their other behavior does not support this hypothesis. We believe that such a trend might be attributed to lack of insurance company support. We suggest that the health system should encourage insurance companies to provide for lab tests requested by dentists too.

## Conclusions

Although our participants were medical specialists in various fields and were afraid of acquiring HBV and HCV, their knowledge about routes of transmission, prevalence, protection and post-exposure seroconversion rates of both HBV and HCV was unsatisfactory. As our participants’ health is not just their own concern and their possible infection and underestimation of HBV and HCV infection could be a threat for their patients, their close contacts and society, more thoroughgoing education is needed. Providing post-exposure prophylaxis facilities could also increase the reporting of needlestick injuries.

This study indicates that our medical specialists are not alert enough to HBV and HCV. They must be sufficiently well informed to be able to improve knowledge, attitudes and behavior of other HCWs and patients. It also seems evident that additional research on HCV is needed in this regard.
